# Underweight and Predictors Among Children Aged 6–59 Months in South Ethiopia

**DOI:** 10.3389/ijph.2024.1606837

**Published:** 2024-05-21

**Authors:** Temesgen Mohammed Toma, Kassahun Tamene Andargie, Rahel Abera Alula, Bahiru Mulatu Kebede, Kidus Temesgen, Tamirat Gezahegn Guyo

**Affiliations:** ^1^ Department of Public Health, Arba Minch College of Health Sciences, Arba Minch, Ethiopia; ^2^ Department of Public Health Emergency Management, South Ethiopia Region Public Health Institute, Jinka, Ethiopia; ^3^ Department of Nursing, Arba Minch College of Health Sciences, Arba Minch, Ethiopia; ^4^ School of Public Health, College of Medicine and Health Sciences, Arba Minch University, Arba Minch, Ethiopia

**Keywords:** underweight, predictors, children, south Ari district, South Ethiopia

## Abstract

**Objectives:**

Underweight remains the primary public health concern among under-five-year-old children in Ethiopia, despite numerous government-implemented nutrition-specific and sensitive interventions. Therefore, this study aimed to assess underweight and associated factors among children in South Ethiopia.

**Methods:**

Between March and April 2021, 717 children in the South Ari district who were between the ages of 6 and 59 months participated in a cross-sectional study. To choose a sample of households containing a minimum of one child between the ages of 6 and 59 months, a multi-stage sampling procedure was used. Data were collected by interviewer-administered structured questionnaires from the mothers of the children. To determine the factors associated with being underweight, a binary logistic regression analysis was built. All variables with a *p*-value <0.25 in the bivariable analysis were entered into a multivariable logistic regression analysis. To assess the strength of the association, an adjusted odds ratio (AOR) with a 95% confidence interval was used. With a *p*-value below 0.05, statistical significance was declared.

**Results:**

The prevalence of underweight was determined to be 29.7% (95% CI: 26.5%, 33.2%). Child age 6–23 months [adjusted odds ratio (AOR) = 0.28; 95% confidence interval (CI): 0.18, 0.44], food insecurity (AOR = 1.48; 95% CI: 1.04, 2.10), sub-optimal child dietary diversity (AOR = 1.44; 95% CI: 1.01, 2.03), birth interval <24 months (AOR = 2.49; 95% CI: 1.75, 3.54), and common childhood illness (AOR = 2.21; 95% CI: 1.52, 3.21) were associated with being underweight.

**Conclusion:**

Underweight among children is predicted by household food insecurity, suboptimal dietary diversity, and common childhood illnesses, necessitating further efforts to improve food security and manage common illnesses.

## Introduction

Undernutrition is a type of malnutrition that results from deficiencies in an individual’s intake of energy and deficiencies in vitamins and minerals, and it includes being underweight, stunting, and wasting. Undernutrition makes under-five children much more susceptible to morbidity and mortality [1]. Being undernourished during childhood may continue up to preschool age and adulthood and result in poor school performance and weak immune systems [2]. Underweight is characterized as a lower weight-for-age, an indicator of the overall population’s nutritional status, and is a combination of both stunting and wasting [3].

Even though the prevalence of underweight among children less than 5 years of age decreased globally between 1990 and 2019, from 24.8% to 13%, it remained a significant public health issue [4]. Globally, 149 million children under five were projected to be stunted and forty-five million to be wasted in 2020 [1]. In Sub-Saharan African countries about 39.9% of children under 5 years who are affected by undernutrition are underweight and this is the highest burden of underweight among these age groups [5]. Evidence from Ethiopian Demographic and Health Surveys (EDHS) revealed that the occurrence of underweight decreased from 33% in 2011 to 21% in 2019 [6]. In Ethiopia, different previous studies revealed different proportions for underweight extending from 5 percent to 32 percent [7, 8].

Underweight has a substantial effect on the health, development, and wellbeing of children whose age is less than 5 years [9]. Children, who experienced undernutrition, were at high risk of reduced cognitive development which leads to poor academic performance, reduced school admission, and absenteeism; this in turn results in abridged productivity during the time of adulthood [10]. According to evidence from the World Health Organization (WHO), malnutrition is considered the single most important risk to the health of the world’s population [11].

Evidence from previous studies revealed that several factors contributed to being underweight among 6–59 months-aged children. Factors including child age, maternal education, family size, gender, maternal occupation, household wealth index, food insecurity, and poor child dietary diversity were associated with being underweight [12–16].

To alleviate this significant public health problem, different interventions were applied. Ethiopia’s government aspires to guarantee that all people have year-round access to safe, nutritious foods and to eradicate all types of malnutrition. Ethiopia has adopted some Nutrition-Sensitive Agriculture [17] measures to enhance children’s and women’s nutritional status by improving the quality and quantity of available, accessible, and inexpensive food and promoting the use of nutritious, diverse, and safe foods for all citizens every time [18]. South Ari Zone is a home to pastoralists, semi-pastoralists, and agrarians. South Ari district is among the areas where agrarians live. According to the zone’s report, malnutrition (acute and chronic) among children was the leading cause of admission to health facilities in the district even though the district was known for having a high vegetable, fruit, and crop production when compared to the zone’s semi-pastoralist and pastoralist districts [19, 20].

Despite the solutions tried, the problem is still evident and studies recommended frequent research to understand the trend of the prevalence of underweight and associated factors [21]. Therefore, the purpose of this study was to determine the prevalence of underweight among children between the ages of six and 59 months in the South Ari district, South Ethiopia, as well as the associated factors.

## Methods

### Study Area, Period, and Design

A community-based cross-sectional survey was conducted in the South Ari district from 11 March 2021 to 11 April 2021. South Ari district is 767 km from Addis Abeba, the country’s capital, and 17 km from Jinka, the administrative center of the South Ari hub. The district has 31 kebeles (small administrative units or neighbourhoods), out of which 2 are Kolla (lowland), 23 are Woina dega (mid-highland), and 6 are Dega (highland) [20]. The Kolla represents areas below 1,500 m, the Dega includes highlands over 2,300 m, and the Woina-Dega includes areas between 1,500 and 2,300 m. The district’s predicted population for 2020 is 160,896, with 25,121 children under the age of five, according to the Ethiopian Census 2007. There are 22,429 children aged between six and 59 months among the under-five population. The district is primarily rural and economically dependent on agriculture, with important crops including grains, pulses, cassava, fruits, sweet potatoes, and false banana. The main cash crops in the area are teff, maize, and fruits [20].

### Population

All of the South Ari district’s households with children between the ages of 6 and 59 months were included as the source population. All the households in the district that were randomly selected and had children between the ages of 6 and 59 months and who had lived in the study setting for at least 6 months were included in the study. Children diagnosed with severe acute malnutrition, those enrolled in a therapeutic feeding program, and mothers and other primary caregivers who were unable to participate in an interview owing to illness were all excluded from the study.

### Sample Size Determination and Sampling Procedure

The sample size was determined using the single population proportion formula by considering the following assumptions: a 95% level of confidence with a confidence level of Za/2 = 1.96, a prevalence of underweight of 27.6% taken from a study done in Dessie, Northwest Ethiopia [22], and a 4% margin of error. Using the above assumptions and a 1.5 design effect, the sample size was calculated to be 720, and after adding a 5% non-response rate, the final determined sample size used to conduct the study was 756.

**FIGURE 1:**
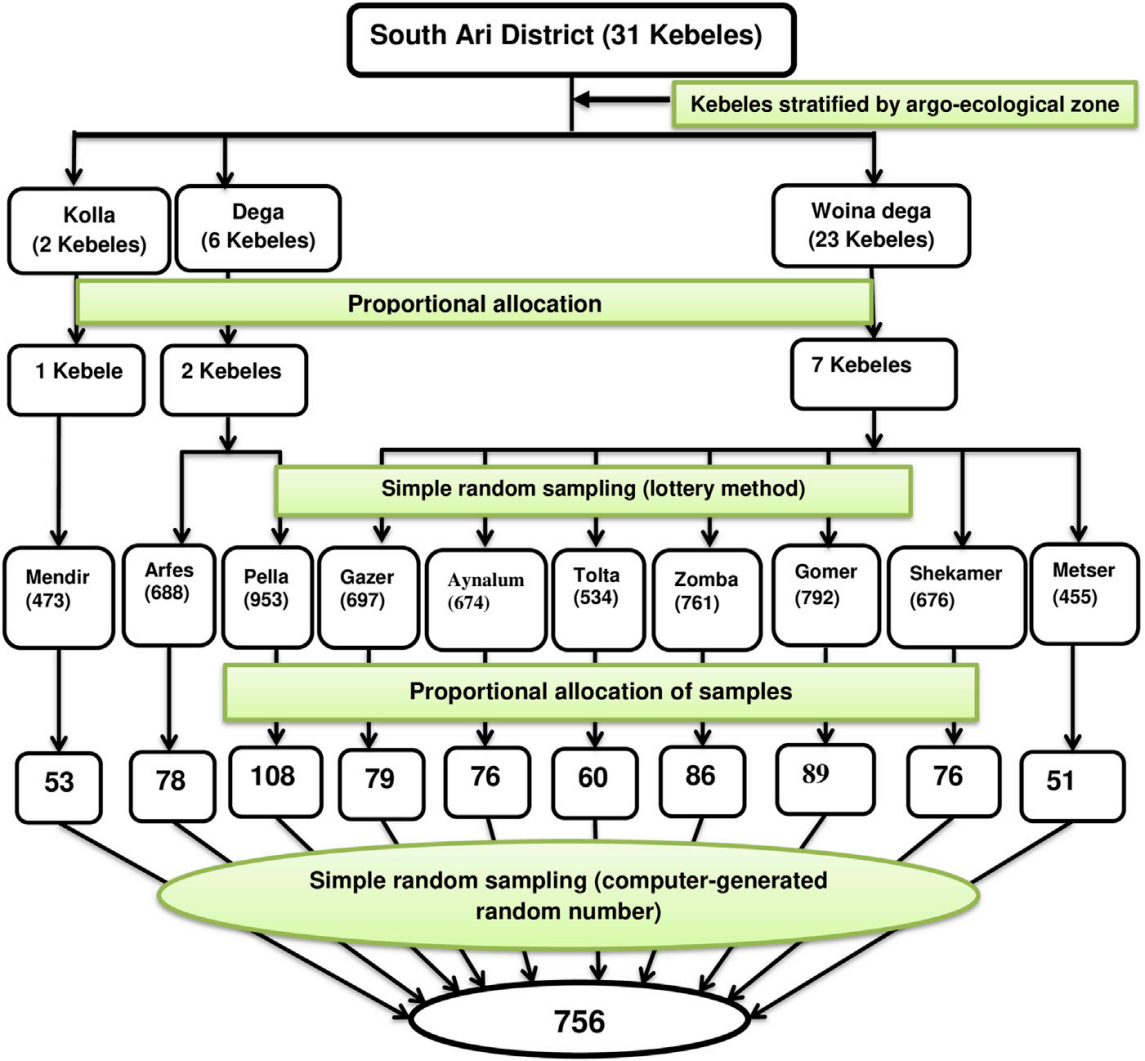
Schematic representation of the sampling procedure to assess underweight and associated factors among children aged 6–59 months in South Ari District, South Ethiopia, 2021: A community-based cross-sectional study.

The study subjects were chosen using a multi-stage sampling process. First, 31 kebeles in the district were classified as Kolla (*n* = 2), Woina Dega (*n* = 23), and Dega (*n* = 6) agroecological zones. Then, using the lottery approach, ten kebeles (one from Kolla, seven from Woina Dega as well as two from Dega) were chosen for the study. The family folder was used to obtain information on households having children aged six up to 59 months. A list of eligible households was created for each of the kebeles and then exported into the Emergency Nutrition Assessment software for random selection. The number of households from each kebele that were included in the study was determined by a proportional allocation to size based on the total number of eligible households living in the kebele. Only one child was chosen using the lottery procedure in selected households with more than one eligible child (Figure 1).

### Study Variables

Underweight among 6–59 month-old children was the dependent variable. Socio-demographic and socio-economic variables such as sex, age, family size, marital status, household wealth index, mother’s employment status, mother’s educational status, household food security status, and father’s education; maternal characteristics like maternal knowledge on child feeding, antenatal care visit, and place of delivery; and child factors like colostrum feeding, complementary feeding initiation age, time of breastfeeding initiation, bottle feeding, pre-lacteal feeding, dietary diversity, non-exclusive breastfeeding, immunization, birth order, birth interval, common childhood illness (fever, diarrhoea, cough) were independent variables.

### Data Collection Procedure and Instrument

A structured tool was developed first in English before being translated into Amharic, and finally back into English by many translators to check consistency. The data collection tool contains socio-economic, socio-demographic, household food insecurity, maternal and child factors, and anthropometric measurements. Training for data collectors and supervisors included a demonstration of anthropometric measurements for 2 days. Data collection including anthropometric measurements was conducted by ten experienced diploma nurses and one health extension practitioner from each selected Kebele. The data collection process was supervised by 2 health officers. Data were collected from the mothers of the children but if the child had no mother then the head of the households were interviewed.

### Measurements and Operational Definitions

#### Household Food Insecurity Measurement

Food security status: Was measured using the Household Food Insecurity Access Scale and categorized as food secure or food insecure. Food secure: If the household experienced none of the food insecurity (access) conditions or just experienced worry, but rarely, whereas food insecure if households are mildly, moderately, or severely food insecure. Households are categorized as mildly food insecure if they worry about not having enough food sometimes or often, and/or households are unable to eat preferred foods, and/or they eat a more monotonous diet than desired, and/or some foods are considered undesirable, but only rarely, and they do not experience the three most severe conditions (running out of food, going to bed hungry, or going a whole day and night without eating), while households with moderate food insecurity tend to compromise quality more frequently by eating uninteresting foods or a monotonous diet sometimes or often, as well as by beginning to reduce their meal size or frequency, rarely or sometimes. But it does not experience one of the three severe conditions. Severely food insecure: any household that experiences one of these three conditions even once in the last 4 weeks (30 days) is considered severely food insecure or has experienced cutting back on meal size or the number of meals often, and/or experiences any of the three most severe conditions (running out of food, going to bed hungry, or going a whole day and night without eating), even as infrequently as rarely [23, 24].

#### Anthropometric Measurement

Children were dressed simply and without shoes while having their weight measured using a portable Seca digital balance to the nearest 0.1 kg. For younger children (6–23 months), the mother and kid were weighed together, the mother individually, and then the child’s weight was calculated by using the tare function to deduct the mother’s weight from the total weight. Before weighing each infant, the scale was adjusted by turning it to zero. Additionally, a known-weight object was used every day to check the weighing scale’s accuracy. The technical error of measures was calculated to reduce anthropometric measurement mistakes. Ten children’s weights were measured twice each to determine the technical error of measurement (TEM) by the primary investigator, who also allowed the data collectors to measure each child twice. Then, a computer program called Emergency Nutrition Assessment (ENA for SMART) was employed.

Underweight was measured by weight-for-age and if children’s weight-for-age is < −2 standard deviations of the WHO Child Growth Standards median the child was classified as underweight [25].

##### Child Dietary Diversity

A 24-h dietary recall method was employed to assess child dietary diversity score (DDS) by using a standardized tool containing seven food groups (grains, roots, and tubers, legumes and nuts, dairy products, flesh foods, eggs, vitamin A rich fruits and vegetables, and other fruits and vegetables). Accordingly, the mothers of the children were asked to report the food types consumed by the child in the last 24 h before the data collection date. If the child consumed four or more of the seven food groups, it was regarded as optimal DDS; otherwise, the child was considered to have sub-optimal DDS [26].

##### Maternal Dietary Diversity

Standardized tool containing ten defined food groups (grains, white roots, and tubers, plantains, pulses, nuts and seeds, dairy, meat, poultry, fish, eggs, dark green leafy vegetables, other vitamin A-rich fruits and vegetables, other vegetables, and other fruits) using 24-h recall method. Accordingly, the mother was asked to report the consumed food group in the previous 24 h. If the mother consumed a minimum of five out of ten defined food groups in the earlier day or night, then the mother was considered to have a high diversity score; otherwise, the mother was considered to have Low DDS [27].

#### Wealth Index

Using easily obtainable information on a household’s possession of a certain 26 types of assets, the wealth index was created [28]. It was produced using principle components analysis (PCA), a statistical technique. The wealth index measures the relative wealth of households, with each asset scored using a factor from PCA. Asset scores normalized to a mean of 0 and standard deviation of 1 were used to create breakpoints to divide the wealth index into poor, medium, and rich categories.

#### Maternal Knowledge of Child Feeding

Assessed using 12-item questions having yes/no responses. A score of one was given for each correctly answered question and a score of zero for each wrong one. Mothers who scored beyond the mean (six) were categorized as having “adequate knowledge,” while, those who scored the mean and lower were categorized as having “inadequate knowledge” [29, 30].

### Data Processing and Analysis

The collected data were checked, entered into Epi-Data 3.1, and then exported to STATA version 14.0 for further management and analysis. According to each variable’s kind, descriptive statistics like; frequency, proportion, mean, median, and standard deviations were computed. After checking the assumptions, the wealth index was computed using PCA and ranked by tertile. To determine underweight status, anthropometric measurements were converted to weight-for-age z-score (WFA) by WHO Anthro software version 3.2.2 using the WHO 2006 child growth standard.

To determine the association between underweight and independent variables, a binary logistic regression analysis model was fitted. All variables with a *p*-value <0.25 in the bivariable analysis were entered into a multivariable logistic regression analysis. Multicollinearity was checked using the variance inflation factor and tolerance. The maximum observed value of the variance inflation factor was 1.03, showing no risk of collinearity. A backward stepwise method was used to fit the final multivariable logistic regression model. At a *p*-value below 0.05, statistical significance was declared. To assess the significance of the association, a 95% confidence interval and an adjusted odds ratio (AOR) were used. To check the goodness of fit of the model, the Hosmer-Lemeshow test statistics (*p*-value = 0.21) were used.

## Results

### Socio-Demographic and Economic Characteristics

A 95% response rate was attained from the 717 respondents who were successfully questioned. Of the study participants, 385 (53.7%) children were male, and 499 (69.6%) children were between the ages of 24 and 59 months. In terms of wealth index, nearly one-third of children came from low-wealth index households. Regarding food security status, 55.2% of children came from food-secure households while 8.6%, 16.3%, and 19.8% of the children who participated in the study were from households with severe, moderate, and mild food insecurity, respectively. More half (55.1%) of the household heads were aged 25–34 years, and 406 (56.6%) of the household heads were men. Among household heads, 164 (22.9%) had not attended formal education. The majority of the mothers, 624 (87%), were married, and 525 of them—or 73.2%—were protestant. Regarding ethnicity, 650 (90.7%) of the participants were Ari by ethnicity, and nearly 42% of the mothers did not attend formal education. Three hundred-seventeen (44.2%) of the mothers were farmers as well as 241 (33.6%) mothers aged 25- to 29-year-old. The majority of the male respondents were farmers 485 (67.9%), and 155 (21.7%) of the fathers had not attended formal education. Ninety (12.6%) of the respondents had families with eight or more members (Table 1).

**TABLE 1:** Socio-demographic and economic characteristics of children (6–59 months) in South Ari District, South Ari Zone, South Ethiopia, 2021: A community-based cross-sectional study (*N* = 717).

Variables		Frequency (N)	Percent (%)
Child Age	6–23 months	218	30.4
	24–59 months	499	69.6
Sex of child	Male	385	53.7
	Female	332	46.3
Household head age (in years)	15–24	32	4.5
	25–34	395	55.1
	≥35	290	40.4
Household head educational status	No formal education	164	22.9
	Primary education	335	46.7
	Secondary education and above	218	30.4
Maternal Age (in years)	15–19	2	0.3
	20–24	151	21.1
	25–29	241	33.6
	30–34	140	19.5
	≥35	183	25.5
Marital status	Single	70	9.8
	Married	624	87.0
	Widowed	8	1.1
	Divorced	15	2.1
Religion	Orthodox	171	23.8
	Protestant	525	73.2
	Muslim	10	1.4
	Catholic	7	1.0
	Others*	6	0.6
Ethnicity	Ari	650	90.6
	Amhara	63	8.8
	Woliata	2	0.3
	Goffa	2	0.3
Maternal education	No formal education	300	41.8
	Primary education	286	39.9
	Secondary education and above	131	18.3
Maternal occupation	Farmer	317	44.2
	Government employee	278	38.8
	Daily labourer	34	4.7
	Merchant	51	7.1
	No work	30	4.2
	Others**	7	1.0
Father’s education (*n* = 714)	No formal education	155	21.7
	Primary education	332	46.5
	Secondary education and above	227	31.8
Father’s occupation (*n* = 714)	Farmer	485	67.9
	Government employee	94	13.2
	Daily labourer	60	8.4
	Merchant	63	8.8
	No work	12	1.7
Family size	2–4	309	43.0
	5–7	318	44.4
	≥8	90	12.6
Household Wealth Index	Poor	239	33.3
	Medium	261	36.4
	Rich	217	30.3
Food security status	Food secure	396	55.2
	Food insecure	321	44.8
Degree of food security status	Food secure	396	55.2
	Mildly food insecure	142	19.8
	Moderately food insecure	117	16.3
	Severely food insecure	62	8.7

Note: *Only Jesus, Pagan; **Housewife, Student.

### Nutrition-Related Characteristics

Of the children, 128 (17.9%), 136 (19.0%), and 112 (15.6%) reported having diarrhoea, cough, and fever in the 2 weeks before the study, respectively. More than ninety percent of children (660 – or – 92.1%) and (684 – or – 95.4%), began to breastfeed timely and were given colostrum, respectively. About one-third of children (27.9%) received non-exclusive breastfeeding, whereas 118 (16.5%) received pre-lacteal feeding. More than nine in ten children, 668 (93.3%), started complementary feeding 6 months and above, while 238 (33.2%) of them were still breastfeeding. More than ninety percent of children (659 – or – 91.9%), were vaccinated, and 351 (49.0%) of the mothers bottle-fed their infants. More than half of the children—384 (53.6%)—had birth intervals of 24 months or longer, and more than a quarter—193 (26.9%)—had birth orders of 4 or higher. Nearly two-fifths (42.8%) of the children had suboptimal dietary diversity scores, according to the study. The majority of mothers—654 or 91.2%—had delivered their child at a healthcare facility, and about eighty percent of them got four or more antenatal care checkups. Out of the respondents, 613 (85.5%) mothers had adequate knowledge of child-feeding practices. A high dietary diversity score of the mother was achieved by 532 (74.2%) of the participants or nearly three-fourths of them (Table 2).

**TABLE 2:** Maternal and child nutrition-related characteristics in South Ari District, South Ethiopia, 2021: A community-based cross-sectional study (*N* = 717).

Variables		Frequency (N)	Percent (%)
Antenatal care follow-up	No	30	4.2
	1–3	116	16.2
	≥4	571	79.6
Place of delivery	Home	63	8.8
	Health institution	654	91.2
Maternal knowledge on child feeding practice	Adequate	613	85.5
	Inadequate	104	14.5
Maternal dietary diversity score (MDDS)	High MDDS	532	74.2
	Low MDDS	185	25.8
Cough in the past 2 weeks	Yes	136	19.0
	No	581	81.0
Diarrhea in the past 2 weeks	Yes	128	17.9
	No	589	82.1
Fever in the past 2 weeks	Yes	112	15.6
	No	605	84.4
Early initiation of breastfeeding	Yes	660	92.1
	No	57	7.9
Colostrum feeding	Yes	684	95.4
	No	33	4.6
Non-exclusive breastfeeding	Yes	200	27.9
	No	517	72.1
Currently on breastfeeding	Yes	238	33.2
	No	479	66.8
Age at initiation of complementary feeding (*n* = 716)	Before 6 months	48	6.7
	6 months and above	668	93.3
Immunization status	Vaccinated	659	91.9
	Unvaccinated	58	8.1
Birth interval	<24 months	333	46.4
	≥24 months	384	53.6
Birth order	First	220	30.7
	2–3	304	42.4
	4 and above	193	26.9
Child dietary diversity score (DDS)	Optimal	410	57.2
	Sub-optimal	307	42.8

Abbreviations: MDDS, maternal dietary diversity score; DDS, Child Dietary Diversity Score.

### Prevalence of Underweight

**FIGURE 2:**
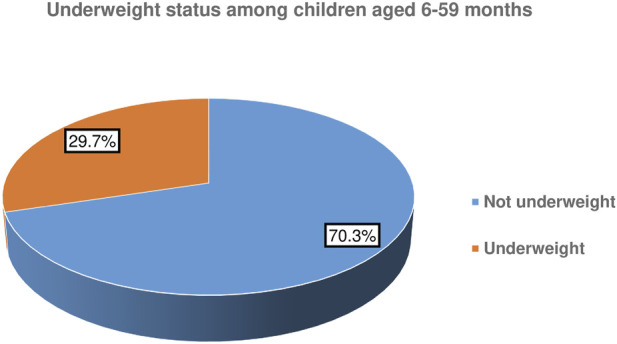
Underweight status of children aged 6–59 months in South Ari district, South Ethiopia, 2021: A community-based cross-sectional study.

This study attested that the prevalence of underweight among children aged six up to 59 months in the study setting was 29.7% (95% CI: 26.5%, 33.2%) (Figure 2).

### Factors Associated With Underweight

In the bivariable logistic regression analysis, the age of the child, sex of the child, marital status, family size, wealth index, food security status, Maternal DDS, antenatal care visit, child DDS, birth interval, age of complementary feeding initiation, bottle feeding, early breastfeeding initiation, immunization status, and common childhood illnesses history were associated with being underweight. In the multivariable logistic regression model, after controlling confounding variables, child sex, age of the child, child DDS, food security status, birth interval, and common childhood illness were factors associated with underweight at *p*-value<0.05. Children residing in food-insecure households have 1.48 times increased odds of being underweight compared to their counterparts (AOR = 1.48; 95% CI: 1.04, 2.10). The likelihood of being underweight among children with sub-optimal dietary diversity is 1.44 times higher compared to those with optimal dietary diversity (AOR = 1.44; 95% CI: 1.01, 2.03). Children with a history of at least one of the common childhood illnesses in the earlier 2 weeks were two times more at risk of being underweight compared to their complements (AOR = 2.21; 95% CI: 1.52, 3.21) (Table 3).

**TABLE 3:** Bivariable and multivariable regression analysis to assess factors associated with underweight among children aged 6–59 months in South Ari district, South Ethiopia, 2021: A community-based cross-sectional study (*n* = 717).

Variables		Underweight status		COR (95% CI)	AOR (95% CI)	*p*-value
		Yes (%)	No (%)			
Age of the household head (in years)	15–24	7 (1.0)	25 (3.5)	0.83 (0.35, 2.00)	0.82 (0.30, 2.24)	0.69
	25–34	133 (18.6)	262 (36.5)	1.51 (1.08, 2.11)	1.32 (0.91, 1.93)	0.14
	≥35	73 (10.2)	217 (30.3)	1	1	
Age of the child (in Months)	6–23	30 (4.2)	188 (26.2)	0.28 (0.18, 0.42)	0.28 (0.18, 0.44)	<0.001
	24–59	183 (25.5)	316 (44.1)	1	1	
Sex of the child	Male	105 (14.6)	280 (39.1)	1	1	
	Female	108 (15.1)	224 (31.2)	1.29 (0.93, 1.77)	1.43 (1.01, 2.02)	0.04
Marital status	In union	192 (26.8)	432 (60.3)	1	1	
	Not in union	21 (2.9)	72 (10.0)	0.66 (0.39, 1.10)	0.72 (0.41, 1.24)	0.23
Family size (in number)	2–4	95 (13.3)	214 (29.9)	1	1	
	5–7	97 (13.5)	221 (30.8)	0.99 (0.70, 1.39)	1.34 (0.89, 2.02)	0.16
	≥8	21 (2.9)	69 (9.6)	0.69 (0.40, 1.18)	0.98 (0.52, 1.85)	0.94
Wealth index	Poor	87 (12.1)	152 (21.2)	1.86 (1.24, 2.81)	1.30 (0.81, 2.08)	0.28
	Medium	75 (10.5)	186 (25.9)	1.31 (0.87, 1.98)	0.97 (0.62, 1.52)	0.89
	Rich	51 (7.1)	166 (23.2)	1	1	
Food security status	Food secure	103 (14.4)	293 (40.9)	1	1	
	Food insecure	110 (15.3)	211 (29.4)	1.49 (1.07, 2.05)	1.48 (1.04, 2.10)	0.03
Maternal dietary diversity score	Low	65 (9.1)	120 (16.7)	1.41 (0.98, 2.01)	1.14 (0.74, 1.75)	0.55
	High	148 (20.6)	384 (53.6)	1	1	
Antenatal care follow-up	<4 times	55 (7.7)	91 (12.7)	1.58 (1.08, 2.31)	1.47 (0.96, 2.24)	0.07
	≥4 times	158 (22.0)	413 (57.6)	1	1	
Child dietary diversity score	Sub-optimal	108 (15.1)	199 (27.8)	1.58 (1.14, 2.18)	1.44 (1.01, 2.03)	0.04
	Optimal	105 (14.6)	305 (42.5)	1	1	
Birth interval	<24 months	132 (18.4)	201 (28.0)	2.46 (1.77, 3.41)	2.49 (1.75, 3.54)	<0.001
	≥24 months	81 (11.3)	303 (42.3)	1	1	
Age of complementary feeding initiation	Before 6 months	8 (1.1)	40 (5.6)	0.45 (0.21, 0.98)	0.61 (0.27, 1.40)	0.24
	6 months and above	205 (28.6)	463 (64.7)	1	1	
Bottle feeding	Yes	94 (13.1)	257 (35.8)	0.76 (0.55, 1.05)	0.79 (0.55, 1.12)	0.18
	No	119 (16.6)	247 (34.5)	1	1	
Early initiation of breastfeeding	Yes	201 (28.0)	459 (64.0)	1	1	
	No	12 (1.7)	45 (6.3)	0.61 (0.32, 1.18)	0.81 (0.39, 1.69)	0.58
Non-exclusive breastfeeding	Yes	46 (6.4)	154 (21.5)	0.63 (0.43, 0.91)	0.74 (0.49, 1.12)	0.15
	No	167 (23.3)	350 (48.8)	1	1	
Immunization status	Vaccinated	190 (26.5)	469 (65.4)	1	1	
	Unvaccinated	23 (3.2)	35 (4.9)	1.62 (0.93, 2.82)	1.46 (0.77, 2.75)	0.24
Common childhood illness	Yes	84 (11.7)	105 (14.6)	2.47 (1.75, 3.51)	2.21 (1.52, 3.21)	<0.001
	No	129 (18.0)	399 (55.7)	1	1	

## Discussion

The prevalence of underweight was 29.7% among children aged 6 upto 59 months in the District. Child age, being female, household food insecurity, children’s sub-optimal dietary diversity, <24 months birth interval, and having common childhood illness were the identified predictors of being underweight.

The prevalence of underweight was determined to be 29.7% by this study. This finding is in line with previously conducted studies in Ethiopia, which reported a prevalence of underweight ranging from 21.7% to 27.6% [15, 22, 31]. This finding is lower than studies done in Bangladesh (43%) [32] and Yemen (46.2%) [33]. On the contrary, the finding of the current study is higher than studies conducted in different regions of Ethiopia, which reported an underweight prevalence ranging from 13.5% to 19.95% [13, 16, 34, 35]. In addition, evidence from the current study is higher than studies done in Vietnam (11.4%) [36], Khyber Pakhtunkhwa (8.4%) [37], and Sub-Saharan Africa (16.3%) [5]. The possible reasons for the disparities between studies might be due to variations in study settings, socio-demographic and economic characteristics, topography as well as cultural differences in the caring and feeding practice of children, and developmental differences. Another likely reason might be that nearly half participants from the current study setting lived in a food-insecure area with a sub-optimal dietary diversity score among children, minimizing the frequency of feeding and making it difficult to provide diversified food. This finding implies that much work is needed to attain the SDG target 2.2 of ending all forms of malnutrition including underweight by 2030.

In this study, it was identified that children aged six upto 23 months had a reduced risk of being underweight compared to those aged 24–59 months. This may be explained by the reason that, as a child gets older they might be put on a family diet and may experience inadequate dietary intake (poor diet), despite increased caloric need for growth and development. Furthermore, as the child gets older, they might be prone to poor sanitation and hygiene and increased risk of infections including intestinal parasites, which might result in the vicious cycle of malnutrition. On the other hand, children aged 6 up to 23 months might be on complementary feeding, and this can help them get a chance to consume more vitamin-A-rich fruits, dairy products, and vegetables which can significantly lower the likelihood of being underweight.

As revealed by this study, sex is another socio-demographic factor that is significantly associated with being underweight. The risk of being underweight is higher among female children than males. The possible reason may be due to the fact that in the current study, there is a higher preference for male sex and families are highly concerned about their male child’s health and nutrition. This might prone female children to be more underweight when compared to their complement.

In this study, household food insecurity is associated with being underweight is household food insecurity. Children from food insecure households had an increased risk of being underweight than their counterparts. This finding is supported by evidence from studies conducted in different parts of Ethiopia: Northern Shewa Zone [16], Sekela District, Western Ethiopia [38], East Badawacho District [39], Semen Bench District [12], Nepal [40], Bangladesh and Vietnam [41]. Furthermore, sub-optimal dietary diversity of children is significantly associated with being underweight. The finding of this study is in line with studies conducted in the Semen Bench district, Ethiopia [12], Tanzania [42], and India [43] which stated that children who consumed less than four of the major food groups from seven food groups were more likely to develop underweight when compared to their complements. Children from food-insecure households might have insufficient access to diversified daily food intakes, which could result in an increased risk of being underweight. Moreover, household food insecurity might affect the availability and utilization of optimal dietary intake and might expose to increased risk of both macro- and micronutrient deficiencies, resulting in malnutrition (underweight).

The current study identified that birth interval is significantly associated with being underweight. This is supported by findings from India [44], China [45], and Sub-Saharan Africa [46]. This finding might be explained by having fewer birth intervals could increase the sharing of food among siblings and affect the quality care and duration of breastfeeding for the index child. Besides, fever birth interval might lead to decreased nutrient reserve for the mother, which exposes to intrauterine growth restriction and being underweight after birth because of the intergenerational effect of malnutrition.

Moreover, having at least one common childhood illness history is associated with being underweight as revealed by this study. The possible reason could be that children with illnesses like diarrhoea, pneumonia, and fever can have reduced apatite, which results in inadequate dietary intake, weight loss, lowered immunity, mucosal damage, and impaired growth and development, which exposes them to being underweight. Malnutrition, in general, can increase a person’s vulnerability to illness, and infection can also contribute to malnutrition, implying a vicious cycle of malnutrition and infection [47].

The finding of this study highlights that policymakers and programmers should give due emphasis to the reduction of malnutrition especially underweight for attaining SDG 3 through comprehensive implementation of interventions addressing identified factors.

The study has some limitations. It might be difficult to see the cause-effect relationship because of the character of the cross-sectional study design. Even though the due emphasis was given to reminding past events by linking them with usual events, there might be some recall bias among participants when assessing common childhood illness, twenty-four-child dietary diversity recall, 1-month household food security recall, and breastfeeding patterns. The standard tools used to assess food insecurity and dietary diversity cannot indicate the seasonal difference that happened over a year. The effect of some important variables like father’s knowledge on child feeding practice. Though emphasis like intensive practical training on anthropometric measurement, pretesting, calibration of instruments, standardizing measurement, computing TEM, and active supervision of field data collection to minimize bias was given, there might be measurement error.

### Policy and Program Implication

The Ethiopian government launched the Seqota Declaration, a high-level commitment to end malnutrition among children by 2030 [48]. Despite this major collaborative platform, the present study finding indicated that the prevalence of underweight was high among children aged 6–59 months due to different factors. The findings of the current study are important for policy, program, and practice in the district, aligning with the second goal of the SDGs, which focuses on nutrition [49] and the World Health Assembly’s (WHA) six global nutrition targets focus on maternal, infant, and young children nutrition to be achieved between 2012 and 2025 [50]. Hence, the finding highlights that policymakers and programmers should give due emphasis to the reduction of malnutrition, especially underweight, for attaining SDG 2 and WHA targets through comprehensive implementation of nutrition-sensitive and specific interventions addressing identified factors. In addition, this public health problem requires integrated and consistent interventions from the district, zonal, regional health bureau, and other stakeholders to achieve the Ethiopian national commitment to end undernutrition among children. Non-governmental organizations need to give due attention to malnutrition screening to detect the underweight as early as possible, and intervention programs to control the prevalence of underweight among children aged 6–59 months. Furthermore, designing health policy should be considered to improve household food security status, ensure consumption of diversified diet among under-five children, and prevent common childhood illnesses that are contributors of underweight to enhance the normal growth of the children.

### Conclusion

In this study, underweight was determined to be a significant public health concern in the district. Child age, being female, household food insecurity, children’s sub-optimal dietary diversity, fewer birth intervals, and having common childhood illnesses were the identified factors of being underweight. Hence, additional effort to improve household food security and feed children a diversified diet is required with due attention given to female children and children 24–59 months. Moreover, strengthening integrated management of common childhood illnesses and child care is highly demanding. More well-designed longitudinal studies are needed to guide efficient interventions that address food insecurity and underweight.
